# HIF-1α Regulates Osteogenesis of Periosteum-Derived Stem Cells Under Hypoxia Conditions *via* Modulating POSTN Expression

**DOI:** 10.3389/fcell.2022.836285

**Published:** 2022-02-17

**Authors:** Yu Zhuang, Zhiyang Zhao, Mengjia Cheng, Meng Li, Jiawen Si, Kaili Lin, Hongbo Yu

**Affiliations:** ^1^ Department of Oral and Cranio-Maxillofacial Surgery, Shanghai Ninth People’s Hospital, College of Stomatology, Shanghai Jiao Tong University School of Medicine, Shanghai, China; ^2^ National Clinical Research Center for Oral Diseases, Shanghai, China; ^3^ Shanghai Key Laboratory of Stomatology and Shanghai Research Institute of Stomatology, Shanghai, China

**Keywords:** periosteum-derived stem cells, hypoxia, HIF-1α, POSTN, bone regeneration

## Abstract

Periosteum is indispensable in bone repair and is an important source of skeletal stem cells (SSCs) for endogenous bone regeneration. However, there are only a few studies about SSCs in periosteum. The craniomaxillofacial bone regeneration is done under the hypoxia microenvironment, in which HIF-1α plays an important role. The effect of HIF-1α on periosteum-derived stem cells (PDSCs) and the mechanisms of PDSCs activation under hypoxia conditions are unknown. In this study, the calvarial bone defect was established, with the periosteum removed or retained. Results show that the bone regeneration was severely impaired in the periosteum removed group. Moreover, pluripotent PDSCs isolated from the periosteum were positive for mesenchymal stem cell (MSC) markers. To determine the role of HIF-1α, the expression of HIF-1α was knocked down *in vivo* and *in vitro*, impairing the bone regeneration or osteogenesis of PDSCs. Furthermore, the knockdown of HIF-1α expression also reduced periostin (POSTN) expression, and recombinant POSTN addition partly rescued the osteogenic inhibition. Finally, to explore the mechanism under POSTN activation, the phosphorylation level of the PI3K/AKT pathway was assessed in transfected PDSCs. The phosphorylation level of PI3K and AKT was enhanced with HIF-1α overexpression and inhibited with HIF-1α knockdown, and the addition of PI3K activator or AKT activator could partly rescue POSTN expression. In conclusion, as a potential target to promote bone repair under the hypoxia microenvironment, HIF-1α can regulate the osteogenic differentiation of PDSCs *via* the PI3K/AKT/POSTN pathway, which lay a solid foundation for periosteum-based craniomaxillofacial bone regeneration.

## 1 Introduction

Congenital or secondary craniomaxillofacial bone defect is common in clinic, which seriously impairs a patient’s function, facial appearance, and mental health. The reconstruction of bone defects is still a challenge (Quarto et al., 2001). Conventional strategies include homologous or allograft bone grafting, but donor site injury and immune rejection are unavoidable. Fortunately, with the development of stem cells and biomaterials, bone tissue engineering provides us with an ideal alternative for bone regeneration. Seed cell is one of the key elements in tissue engineering and is mainly pluripotent stem cells in which it plays a vital role. Research studying the characterizations of stem cells as seed cell for tissue engineering has received much attention. Moreover, cell-based therapies have also been investigated in intractable diseases and injuries, some of which have been applied in clinical trials (Yamanaka, 2020). Skeletal stem cells (SSCs), distributed in different compartments along endosteum, periosteum, and metaphyseal trabeculae, play an important role in the defect repair process (Colnot et al., 2003; Yu et al., 2010).

The bone development, repair, and regeneration depend on two important ossification processes: endochondral ossification (requiring mineralization in cartilage) and intramembrane ossification (dependent on MSC differentiating into osteoblasts) (Hall, 2005). Compared with endochondral ossification, intramembrane ossification is more common in craniomaxillofacial bone defect. Thus, the performance of periosteum in craniomaxillofacial bone repair is emphasized. Periosteum, the thin layer with vessels, nerves, and cells along the bone surface, is an important SSCs source for endogenous bone regeneration (Zhang et al., 2005; Thabet et al., 2008; Colnot, 2009; Colnot et al., 2012; van Gastel et al., 2012; Lin et al., 2014). Moreover, mesenchymal stem cells (MSCs) in the periosteum may play an indispensable role in bone development. Recent stem cell research has focused on bone marrow mesenchymal stem cells (BMSCs). Despite the wide application, BMSCs are not proven to be the central cellular element in regeneration (Zhang et al., 2003; Sacchetti et al., 2007; Crane and Cao, 2014; Morrison and Scadden, 2014). Additionally, the craniomaxillofacial bone is a flat bone, unlike the long bone, lacking bone marrow tissues and poor in endogenous stem cell recruitment from the whole body, which further emphasizes the importance of periosteum and periosteum-derived stem cells (PDSCs). Despite the significance, the study on the characterization of PDSCs is rare. Additionally, skeletal reprogramming to bone regeneration requires reactivating key transcription and growth factors. However, mechanisms under the approaches to PDSCs activation remain unintelligible.

Because trauma and bone fracture usually impair the local vascular system, the healing process of craniomaxillofacial bone defect is proceeded under the hypoxia microenvironment (Kanczler et al., 2008). It has been shown that in the rabbit fracture model, the oxygen tension in the fracture repair region is only about 1% (Kim et al., 2009). The local hypoxia microenvironment can regulate cytokines synthesis. The biological performance of stem cells under hypoxia can be different from that under normoxia conditions (Zhang et al., 2021). Hypoxia-inducible factor-1α (HIF-1α), a classic transcription factor under hypoxic environment, is degraded under normoxia and is induced only under hypoxia, serving as an important factor to activate stem cells during the initial bone defect repair process and regulate the expression of hypoxia-responsive genes (Taylor and Colgan, 2017; Cirillo et al., 2020; Todd and Johnson, 2020). Studies showed that hypoxia signals and HIF-1α are involved in the osteogenic differentiation of BMSCs (Stegen et al., 2016; Hirai et al., 2018; Zhang et al., 2019). However, the effect and mechanism of HIF-1α on PDSCs differentiation and activation are still unclear.

Herein, we explored the effect of HIF-1α on PDSCs osteogenic differentiation under the hypoxia microenvironment, the key factors in PDSCs osteogenesis regulation, and the potential downstream signal pathway. Furthermore, we addressed the importance of periosteum and PDSCs in craniomaxillofacial bone regeneration.

## 2 Materials and Methods

### 2.1 Animals

Six-week-old and four-week-old C57BL/6 mice and six-week-old SD rats were purchased from the animal center of Shanghai Ninth People’s Hospital, Shanghai Jiao Tong University School of Medicine. The animal experiments were approved by the Ethics Committee of Shanghai Ninth People’s Hospital (Certificate No. SH9H-2021-A280-S8).

### 2.2 Calvarial Defect Model Construction

The rats were randomly separated into two groups. In one group, the calvarial periosteum above the defect was removed and in the other, it was not. Mice (six-week-old) and rats (six-week-old) were firstly anesthetized. In rat calvarial bone defect model construction, skin, subcutaneous tissue, and periosteum were cut and stripped. A 5 mm bone defect was made using an orthopedic drill. In the periosteum retained group, subcutaneous tissue and periosteum are sewed using absorbable suture material. Then, the skin was sewed using a nonabsorbable suture. In the periosteum removed group, the size of the removed periosteum is 5 mm, consistent with the size of the bone defect. After cutting and bone defect preparation, the above periosteum was removed using a scissor, and then subcutaneous tissue and skin were sewed, respectively. In mice’s calvarial bone defect model construction, the surgical region was disinfected, and the incision was made sagittally with periosteum stripped to expose the calvarial bone. Then, a 1 mm subcritical size defect was made using a round bur in mice. The incision was sutured. The mice were randomly divided into two groups. Short hairpin RNA (shRNA) encapsulated in lentivirus were purchased from Gikai Gene Company (Shanghai, China) and utilized to knock down the HIF-1α expression. The two groups were injected with shNC and shHIF on the local surgical region the day after surgery.

### 2.3 Micro-CT Analysis

The mice were euthanized 20 days after surgery, and the rats were 4 and 8 weeks. The samples were fixed in 4% paraformaldehyde. A micro-CT instrument (Quantum GX, PerkinElmer, United States) was utilized to scan the samples and reconstruct the 2D and 3D figures of the calvarial bone.

### 2.4 Hemotoxylin and Eosin and Masson Staining

The fixed tissues were decalcified, embedded in paraffin, and sliced into 5 μm slices. The slices were deparaffinized and rehydrated. Subsequently, HE and Masson staining were conducted according to the manufacturer’s instruction of the HE and Masson staining kit (Solarbio, Bejing, China).

### 2.5 Immunofluorescence

For slice immunofluorescence, the slices were firstly deparaffinized and rehydrated, and antigen retrieval was done. For cell immunofluorescence, cells were fixed with 4% paraformaldehyde. For both slice and cell immunofluorescence, samples were permeabilized, blocked, and incubated in primary antibody rabbit anti-mouse HIF-1α (1:200, Immunoway, Plano, TX), rat anti-mouse CD44 (1:250, Abcam, Cambridge, United Kingdom), rabbit anti-mouse POSTN (1:600, Abcam, Cambridge, United Kingdom) at 4°C overnight. Then, the samples were washed and incubated in Alexa Fluor 555 goat anti-rabbit (1:500, Invitrogen, Waltham, MA) and Alexa Fluor 488 goat anti-rat (1:1000, Abcam, Cambridge, United Kingdom) secondary antibody at room temperature for 1 h. They were stained with DAPI (Beyotime Biotechnology, Shanghai, China) for 10 min. The samples were photographed using confocal laser-scanning microscopy (Leica, Germany), and semi-quantification of intensity was analyzed through Image J.

### 2.6 Primary Culture of Periosteum-Derived Stem Cells

The calvarial PDSCs were isolated and cultured according to earlier protocols (Debnath et al., 2018). The calvarial periosteum tissues from four-week-old mice were stripped with razor blades. The tissues were digested in a mixture of 1 mg/ml collagenase P and 2 mg/ml dispase II (Roche, Basel, Swiss) at 37°C for 1 h, centrifuged to remove the supernatant, and sediment was resuspended in 2 units/ml DNase I at 37°C for 5 min. The mixture was resuspended thoroughly and filtrated using a 70 μm mesh. The filtrated medium was then centrifuged, with the cell pellet obtained, and cultured in α-MEM media supplemented with 10% fetal bovine serum (FBS) at 37°C, under 5% CO_2_. Passage 1 of PDSCs was used for *in vitro* assays.

### 2.7 Flow Cytometry Analysis

Cells were incubated with FITC anti-mouse CD90.2 (Thy1.2), APC anti-mouse CD44, Cy7 anti-mouse CD31, and PE anti-mouse CD45 (Biolegend, CA) to label mesenchymal lineage *via* flow cytometry analysis.

### 2.8 *In Vitro* Multiple Differentiation of Periosteum-Derived Stem Cells

The osteogenic, adipogenic, and chondrogenic induced differentiation kits (OriCell, Cyagen, Guangzhou, China) were utilized to induce multi-potent differentiation of PDSCs, according to the manufacturer’s instructions. The images were recorded using a light microscope.

### 2.9 Small Interfering RNA Transfection and Plasmid DNA

The sequence for siRNA targeted HIF-1α and pDNA was designed and synthesized in Sangon Biotech (Shanghai, China). The siRNA and pDNA transfection were assisted using Lipofectamine 2000 Transfection Reagent (Invitrogen, Waltham, MA) following the manufacturer’s instructions. The cells transfected with siHIF-1α and pHIF-1α were cultured in α-MEM supplemented with 2% fetal bovine serum (FBS) at 37°C under hypoxia environment (1% O_2_, 5% CO_2_) for 48 h after transfection, and the transfection efficiency was checked by qRT-PCR, western blotting, and immunofluorescence.

### 2.10 Alkaline Phosphatase Staining

PDSCs cultured in osteogenic medium (OM) under hypoxia after transfection, with or without recombinant mouse POSTN (Sigma-Aldrich, Darmstadt, Germany), were stained utilizing ALP Assay Kit (Beyotime, Shanghai, China).

### 2.11 RNA Isolation and qRT-PCR

The cells with transfection were cultured in OM under a hypoxia environment for 48 h, with or without POSTN, and cellular RNA was extracted *via* TRIzol (Invitrogen, Waltham, MA), according to the manufacturer’s instructions. The values of OD_260/280_ were appropriate, and cDNA was obtained using the Reverse Transcription Kit (Takara Bio, Shiga, Japan). The primer sequences provided by Sangon Biotech (Shanghai, China) are listed in Supplementary Table S1. The qRT-PCR was conducted under the manufacturer’s instruction of SYBR^®^ reagent (Takara Bio, Shiga, Japan), and GAPDH was utilized as the housekeeper gene to normalize gene expression. The results were analyzed and displayed according to the 2^−ΔΔCt^ strategy.

### 2.12 Protein Extraction and Western Blotting Analysis

Transfected PDSCs were cultured in OM under hypoxia for 24 or 48 h after transfection. For the rescue experiment, PI3K specific activator 740-YP 30 μg/ml (MedChemExpress, NJ) and AKT activator SC79 5 μg/ml (Sangon Biotech, Shanghai, China) were utilized. The total proteins were extracted using RIPA (Thermo Fischer Scientific, Waltham, MA). The proteins were denatured at 100°C for 10 min, separated using SDS-PAGE, and then transferred to the PVDF membrane (Millipore, Billerica, MA). Subsequently, the membranes were blocked with non-fat milk, incubated with primary antibody HIF-1α (1:2000), POSTN (1:3000), rabbit anti-mouse PI3K (1:1000, Cell Signaling, Danvers, MA), rabbit anti-mouse p-PI3K (1:1000, Cell Signaling, Danvers, MA), rabbit anti-mouse AKT (1:3000, Proteintech, Chicago, IL), rabbit anti-mouse pAKT (1:1000, Cell Signaling, Danvers, MA), and rabbit anti-mouse GAPDH (1:10000, Proteintech, Chicago, IL) at 4°C; washed with TBST; and incubated with secondary antibody for 1 h. The membranes were visualized using a chemiluminescence imaging system (Tanon, Shanghai, China) and semi-quantified using Image J.

### 2.13 Statistical Analysis

The data were analyzed *via* one-way analysis of variance (ANOVA) and two-tailed *t*-test using GraphPad 8.0 software, and *p* < 0.05 was considered significant.

## 3 Results

### 3.1 Periosteum Destruction Impairs Calvarial Bone Regeneration

**FIGURE 1:**
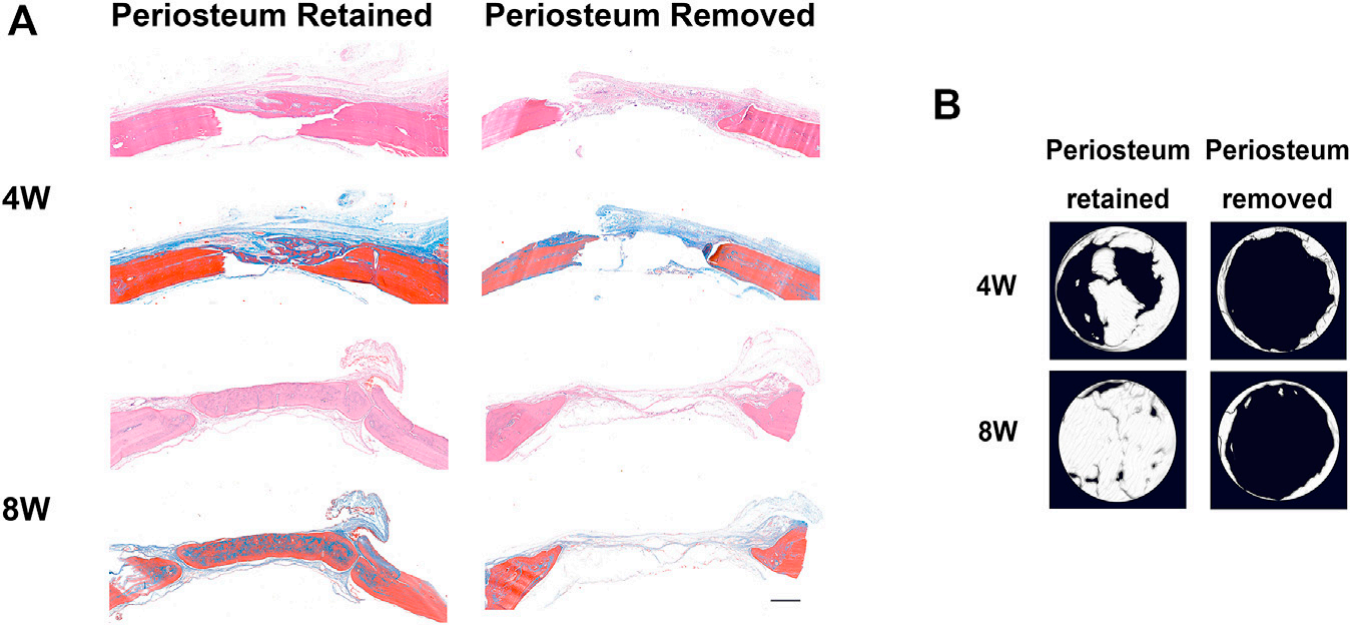
The periosteum is indispensable in rat calvarial bone regeneration. **(A)** HE and Masson staining of defected calvarial bone, with periosteum above the defect region retained or removed, 4 and 8 weeks after surgery (scar bar: 500 μm). **(B)** The micro-CT results for calvarial bone regeneration.

In calvarial bone defect, the periosteum removal severely impaired bone regeneration (Figures 1A,B), which indicated that the periosteum might be indispensable in craniomaxillofacial bone repair.

### 3.2 HIF-1α Is Required for *In Vivo* Bone Regeneration

**FIGURE 2:**
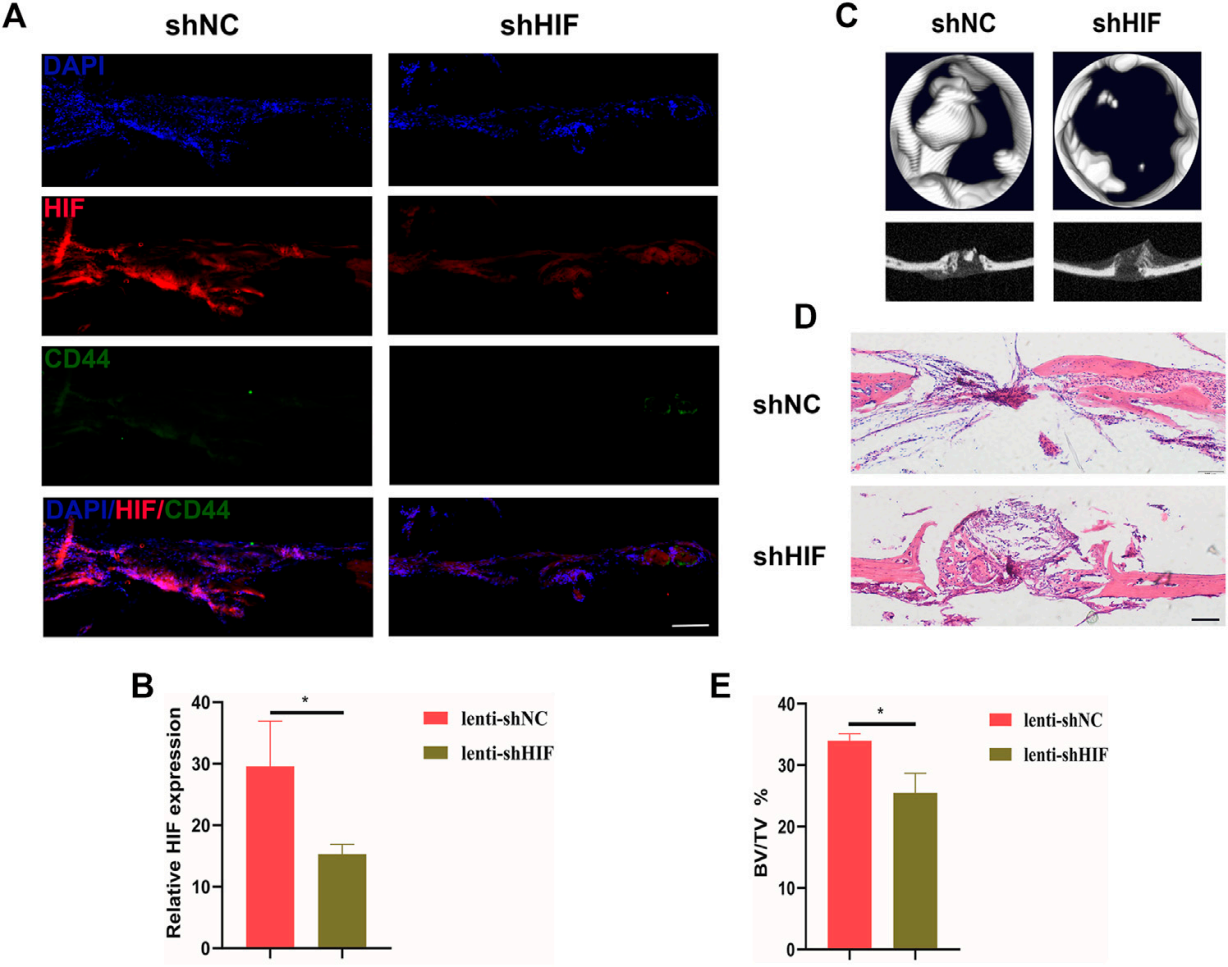
Impaired HIF-1α expression affects *in vivo* bone regeneration of mice. **(A)** The expression of HIF-1α and CD44^+^ cells in the defected region of calvarial bone 20 days after surgery (scar bar: 100 μm). **(B)** Semi-quantification results of HIF-1α expression. **(C)** The 2D and 3D images for micro-CT results. **(D)** HE staining of defected calvarial bone, with shNC or shHIF injection, 20 days after surgery (scar bar: 100 μm). **(E)** The analysis for bone volume/tissue volume (BV/TV) of micro-CT results. * indicates the significance tests between groups: *p < 0.05.

After constructing the calvarial bone defect model, the shHIF was injected into the local surgical region the day after surgery. The slice of the calvarial bone sample showed that the GFP was inserted into the lentivirus expressed 3 days after injection, which indicated the transfection efficiency (Supplementary Figure S1). The HIF-1α expression in the defected region of calvarial bone was knocked down in the shHIF group, compared with the shNC group (Figures 2A,B). The bone regeneration was impaired by the shHIF injection (Figures 2C–E).

### 3.3 HIF-1α Plays an Important Role in Osteogenesis of Periosteum-Derived Stem Cells

**FIGURE 3:**
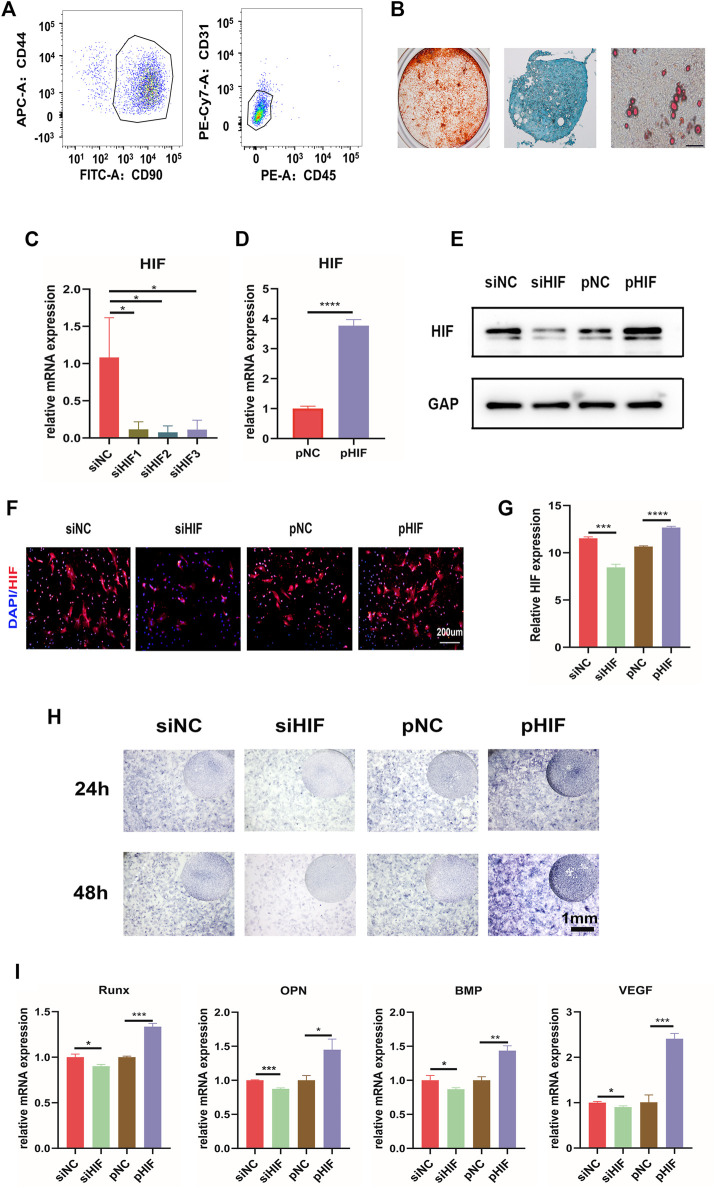
HIF-1α is important in *in vitro* osteogenesis of PDSCs. **(A)** Flow cytometry results of PDSCs isolated from mice calvarial periosteum showing the cells double-positive for CD44 and CD90 (91.1%) and double-negative for CD45 and CD31 (95.9%). **(B)** *In vitro* osteogenic (21D), chondrogenic (14D), and adipogenic (21D) differentiation of PDSCs (scar bar: 100 μm). **(C, D)** The expression of HIF-1α after siHIF-1α and pHIF-1α transfection in PDSCs by qRT-PCR. **(E)** Western blotting of HIF-1α. **(F,G)** The immunofluorescence for HIF-1α in transfected PDSCs and the semi-quantification. **(H)** ALP staining of the PDSCs after transfection. **(I)** The expression of the osteogenic related genes. * indicates the significance tests between groups: *p < 0.05, **p < 0.01, and ***p < 0.001.

CD90 and CD44 were MSC positive markers, and CD45 and CD31 were MSC negative markers. The flow cytometry analysis of adherent cells derived from periosteum showed that the percentage of PDSCs positive for CD90 and CD44 was 91.1%, and that negative for CD45 and CD31 was 95.9% (Figure 3A), which indicated that the isolated PDSCs possess MSC positive markers, without negative markers. Moreover, the osteogenic, chondrogenic, and adipogenic differentiation potential of adherent cells were also verified (Figure 3B), which confirmed that the obtained adherent cells were MSCs. The isolated PDSCs were then utilized for *in vitro* assays. The expression of HIF-1α was knocked down successfully by siHIF transfection and enhanced *via* pHIF transfection (Figures 3C–G). The ALP staining and the expression of osteogenic related genes indicated that under a hypoxia environment, the knockdown of HIF-1α expression inhibited ALP activity and osteogenic related gene expression, while the enhancement was promoted (Figures 3H,I).

### 3.4 HIF‐1α Affects Osteogenesis *via* Modulating POSTN Expression Under Hypoxia Conditions

**FIGURE 4:**
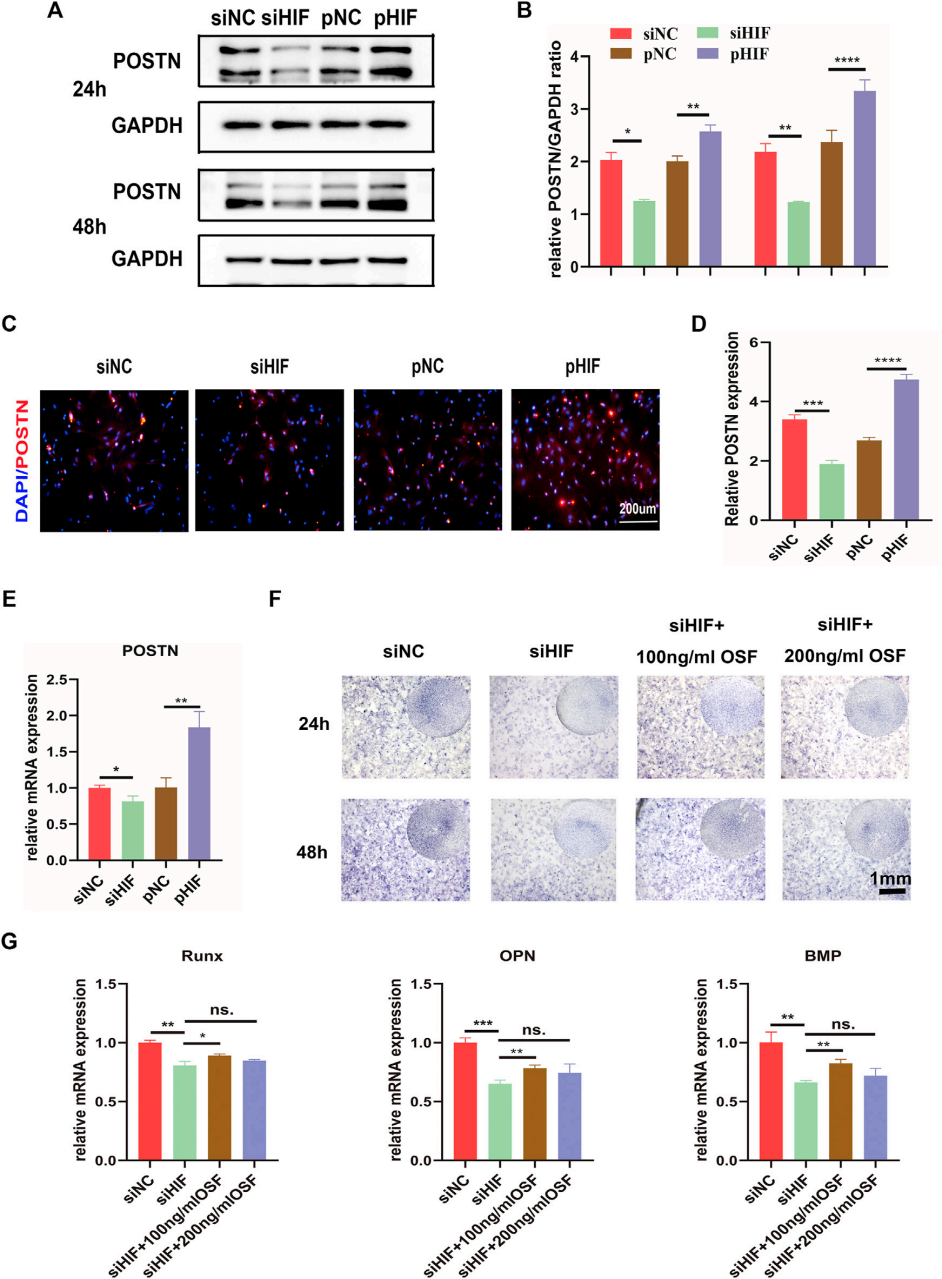
HIF‐1α regulates osteogenesis *via* POSTN. **(A,B)** Western blotting of POSTN expression in PDSCs after siHIF-1α and pHIF-1α transfection and semi-quantification. **(C,D)** The immunofluorescence for POSTN in transfected PDSCs and the semi-quantification. **(E)** The mRNA expression of POSTN by qRT-PCR. **(F)** ALP staining of the PDSCs after transfection, with or without the addition of POSTN. **(G)** The expression of the osteogenic related genes. * indicates the significance tests between groups: *p < 0.05, **p < 0.01, ***p < 0.001, ****p < 0.0001; and ns: no significant difference.

The expression of POSTN was correlated with that of HIF-1α. The knockdown of HIF-1α expression reduced the expression of POSTN, while the enhancement was promoted (Figures 4A–E). To examine whether HIF-1α regulated the osteogenesis *via* POSTN, the effect of POSTN addition into PDSCs transfected with siHIF on osteogenesis was evaluated. The ALP staining and osteogenic related genes expression results indicated that the siHIF would inhibit osteogenesis, and the recombinant POSTN protein addition (100 ng/ml) would partly rescue the osteogenic inhibition. However, the concentration of 200 ng/ml did not make a significant difference (Figures 4F,G).

### 3.5 HIF‐1α Activates POSTN Expression *via* PI3K Signaling Pathway

**FIGURE 5:**
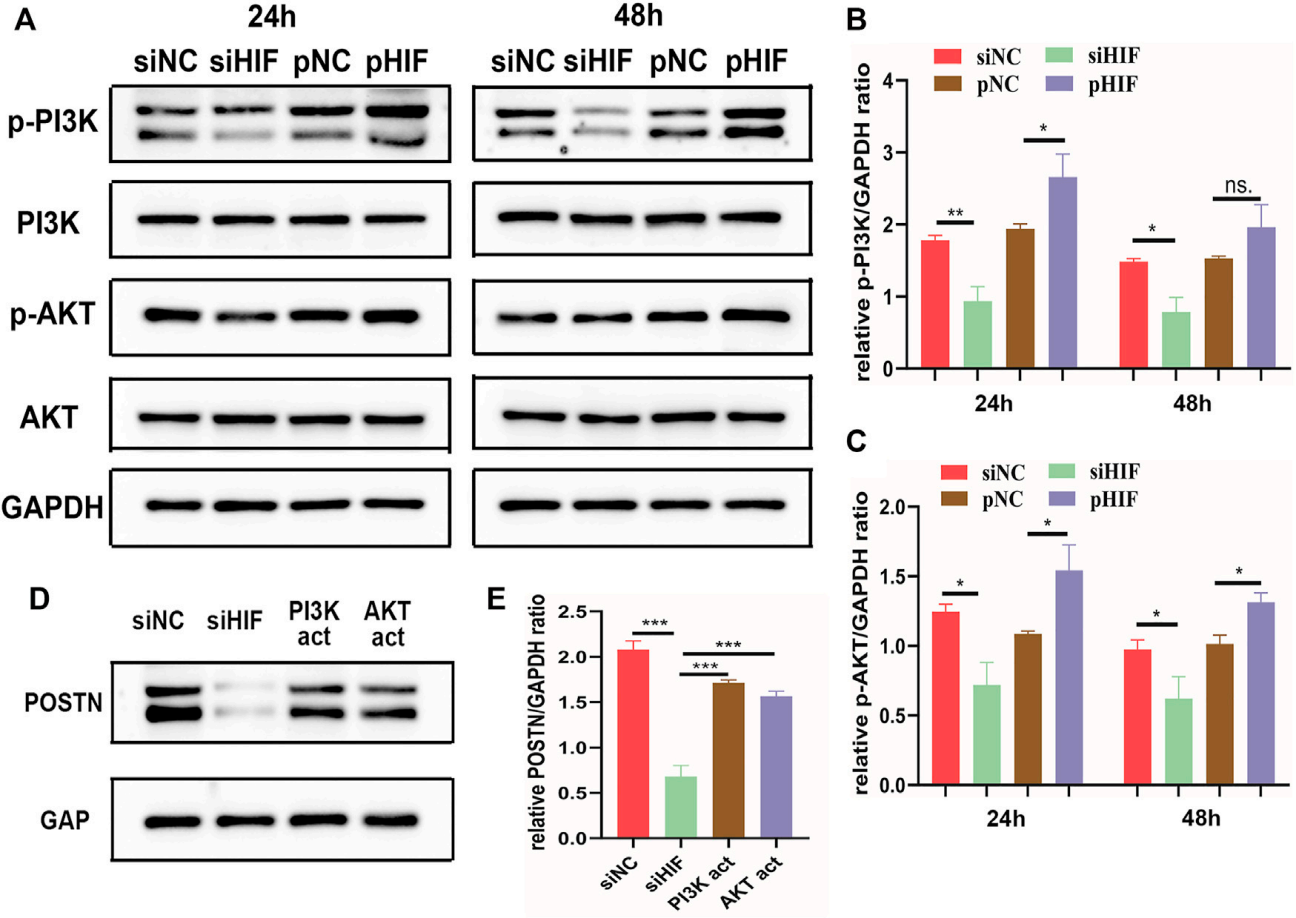
HIF‐1α induces POSTN expression *via* PI3K/AKT signaling pathway. **(A–C)** Western blotting analysis of the phosphorylation of PI3K and AKT in transfected PDSCs and semi-quantification. **(D,E)** Western blotting of POSTN expression in transfected PDSCs in presence or absence of PI3K activator 740-YP (30 μg/ml) or AKT activator SC79 (5 μg/ml), and semi-quantification. * indicates the significance tests between groups: *p < 0.05, **p < 0.01, ***p < 0.001; and ns: no significant difference.

The phosphorylation level of PI3K and AKT was enhanced in pHIF transfected cells and inhibited in siHIF transfected cells (Figures 5A–C). The siHIF transfection in PDSCs inhibited the POSTN expression. However, with the addition of PI3K activator or AKT activator, the POSTN expression was partly rescued (Figures 5D,E), which further indicated that HIF‐1α could induce POSTN expression *via* PI3K/AKT pathway.

## 4 Discussion

In the well-orchestrated process of bone defect repair and regeneration, the activation of SSCs serves as the basic stone. During the bone reconstruction process, the recruitment, adhesion, proliferation, and differentiation of endogenous SSCs are activated. Under the defect repair microenvironment, SSCs differentiate into pre-osteoblasts, express osteogenic-related genes, and secret bone matrix. While the endogenous source and molecular mechanism of SSCs activation are poorly elucidated. During the embryogenesis and ossification process, bone formation starts from the aggregation and coagulation of MSCs. It then progresses to endochondral ossification through cartilage formation or intramembranous ossification through osteoblast-precursors differentiation (Roberts et al., 2015). In the repair process of the long bone, stem cells from the periosteum (periosteum-derived stem cells, PDSCs) are activated and migrated locally, and BMSCs from the bone marrow are stimulated by cytokines and aggregate through blood vessels. At the defect site, they aggregate, proliferate, differentiate, and participate in the formation of new bone (Colnot, 2009). BMSCs are widely applied in cell-based therapies due to their extensive sources and mature preparation technology, while PDSCs have not received widespread attention (Colnot, 2009).

Periosteum plays a crucial role in bone repair and regeneration (Colangeli et al., 2020). In this study, the calvarial bone defect model was established to simulate bone wound healing. The results showed that the bone repair of the periosteum retained group was significantly better than that of the periosteal removal group. Periosteum removal impaired bone repair, indicating the presence of SSCs in the periosteum (Chang and Knothe Tate, 2012; van Gastel et al., 2012). The SSCs were isolated from the periosteum. They verified their three basic characteristics (adherent growth, MSC surface marker, and multi-differentiation potential), thus called periosteum-derived mesenchymal stem cells (PDSCs). The biological performance and mechanism of PDSCs require explanations. Exploring the molecular mechanism under osteogenesis regulation of PDSCs will bring new hope and treatment targets for its application in cell-based therapies and bone tissue engineering.

During the bone defect healing process, hematomas formed firstly around the wound; growth factors are activated; and the recruitment, proliferation, and differentiation of stem cells are activated. Without enough vessels growing into defect regions at the very beginning of the repair period, MSCs play their role under the hypoxia environment. The characteristics of these MSCs may be altered (Fan et al., 2015; Stegen et al., 2019). Lee et al. (2015) found that hypoxia (1% O_2_) culture enhances the osteogenic and chondrogenic of hBMSCs compared with normoxia (21% O_2_) and promotes *in vivo* bone regeneration. Hypoxia-responsive factor (HIF-1α) is indispensable in bone repair under hypoxia (Johnson et al., 2015; Stegen et al., 2016). There have been studies on the effect of HIF-1α on BMSCs, but the influence of HIF-1α on PDSCs is unknown. In our study, knockdown of HIF-1α *in vivo* and *in vitro* significantly impair bone regeneration and osteogenesis of PDSCs, indicating the indispensability of the HIF-1α in bone regeneration under hypoxia. Because HIF-1α is degraded by proline hydroxylase under normoxia (Salceda and Caro, 1997), all *in vitro* experiments in this study are under the hypoxia microenvironment to ensure the activity of HIF-1α and simulate the hypoxia environment in the bone defect.

The PDSCs and BMSCs possess various characteristics. Duchamp de Lageneste et al. (2018) found that the expression of periostin (POSTN) in PDSCs was significantly higher than that in BMSCs through high-throughput analysis of transcriptome RNA isolated from PDSCs and BMSCs responding to fracture defects. POSTN is a highly conserved matrix protein, expressed in the periodontal ligament, periosteum, tendon, and other tissues, as well as in bone tissue. It can activate osteogenesis of osteoblast-precursors through integrin receptors and Wnt-β-catenin signaling pathway (Baron and Kneissel, 2013; Bonnet et al., 2016; Idolazzi et al., 2017). Knockout of the *POSTN* gene in mice resulted in the significantly weakened migration and osteogenesis ability of PDSCs in bone repair (Duchamp de Lageneste et al., 2018), indicating POSTN plays an important regulatory role in promoting the osteogenesis of PDSCs (Gao et al., 2019). HIF-1α might be the upstream gene of POSTN. Zhang et al. (2014) found that the expression of POSTN was positively correlated with that of HIF-1α, and silencing HIF-1α reduced POSTN expression, which indicated that HIF-1α could upregulate the expression of POSTN under hypoxic conditions, thereby modulating the osteogenic ability of PDSCs.

In this study, knockdown the expression of HIF-1α reduced POSTN expression. With the addition of recombinant POSTN, the impaired osteogenic differentiation of PDSCs by siRNA was partly rescued, which indicated that HIF-1α could regulate the osteogenesis of PDSCs *via* POSTN. Moreover, PI3K/AKT signal pathway and POSTN expression were inhibited in siRNA transfected PDSCs, and the activation of PI3K or AKT could enhance the POSTN expression. Thus, it can be deduced that PI3K/AKT might be the signal pathway linking HIF-1α and POSTN.

## 5 Conclusion

Periosteum and PDSCs are important in the craniomaxillofacial bone repair process, and HIF-1α plays an indispensable role in osteogenesis under the hypoxia microenvironment. Moreover, HIF-1α can regulate the osteogenic differentiation of PDSCs *via* the PI3K/AKT/POSTN pathway. The findings indicate that PDSCs are emerging seed cells for bone tissue engineering, and HIF-1α serves as a potential target to promote bone repair, which lays a solid foundation for the periosteum-based craniomaxillofacial bone regeneration.

## Data Availability

The original contributions presented in the study are included in the article/Supplementary Material, further inquiries can be directed to the corresponding authors.
