# Supplementary material for: HIF-1α Regulates Osteogenesis of Periosteum-Derived Stem Cells Under Hypoxia Conditions via Modulating POSTN Expression
Source: Front Cell Dev Biol. 2022 Feb 17;10:836285. doi: 10.3389/fcell.2022.836285 (PMC8891937; doi:10.3389/fcell.2022.836285)
Supplement: Supplementary file 1 [file DataSheet1.docx]

**HIF regulates osteogenesis of periosteum-derived mesenchymal stem cells under hypoxia conditions via modulating POSTN expression**

Supplementary Table 1. Primers for qRT-PCR

| name | Primers |
| --- | --- |
| GAPDH | 5’-AGGTCGGTGTGAACGGATTTG - 3’ |
|  | 5’-TGTAGACCATGTAGTTGAGGTCA - 3’ |
| HIF-1α | 5’-TCAAGTCAGCAACGTGGAAG- 3’ |
|  | 5’-ATCGAGGCTGTGTCGACTG - 3’ |
| POSTN | 5’-TGGTATCAAGGTGCTATCTGCG - 3’ |
|  | 5’-AATGCCCAGCGTGCCATAA - 3’ |
| Runx | 5’-TTACCTACACCCCGCCAGTC - 3’ |
|  | 5’-TGCTGGTCTGGAAGGGTCC - 3’ |
| OPN | 5’-CCCGGTGAAAGTGACTGATT - 3’ |
|  | 5’-TTCTTCAGAGGACACAGCATTC - 3’ |
| BMP2 | 5’-AGATCTGTACCGCAGGCACT - 3’ |
|  | 5’-GTTCCTCCACGGCTTCTTC - 3’ |
| VEGF | 5’-AGGCTGCTGTAACGATGAAG- 3’ |
|  | 5’-TCTCCTATGTGCTGGCTTTG - 3’ |

Supplementary figure 1


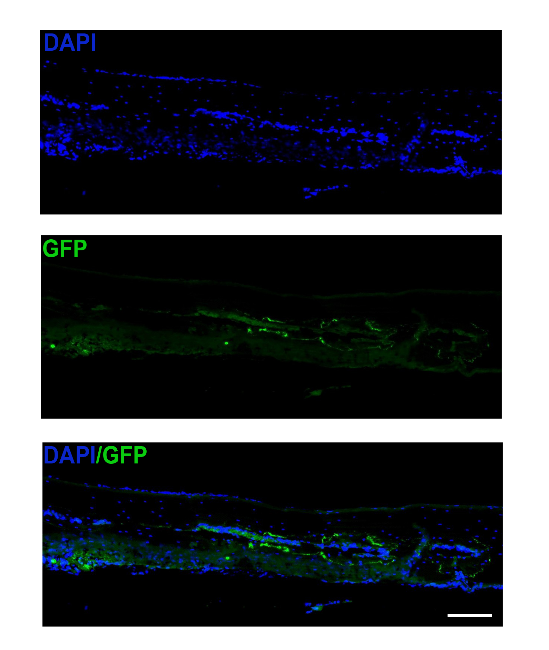


Supplementary fig. 1. The GFP expression 3 days after injection. The calvarial bone sample was obtained, and the expression of GFP inserted into the lentivirus could be observed 3 days after shHIF injection.
